# A political economy analysis of human resources for health (HRH) in Africa

**DOI:** 10.1186/s12960-016-0137-4

**Published:** 2016-07-22

**Authors:** John Vincent Fieno, Yoswa M. Dambisya, Gavin George, Kent Benson

**Affiliations:** 1 Regional HIV/AIDS Program, U.S. Agency for International Development, Pretoria, South Africa; 2 East, Central and Southern Africa Health Community, Arusha, Tanzania; 3 Health Economics and HIV/AIDS Research Division (HEARD), University of KwaZulu-Natal, Durban, South Africa

## Abstract

**Background:**

Despite a global recognition from all stakeholders of the gravity and urgency of health worker shortage in Africa, little progress has been achieved to improve health worker coverage in many of the African human resources for health (HRH) crisis countries. The problem consists in how policy is made, how leaders are accountable, how the World Health Organization (WHO) and foreign donors encourage (or distort) health policy, and how development objectives are prioritized in these countries.

**Methods:**

This paper uses political economy analysis, which stems from a recognition that the solution to the shortage of health workers across Africa involves more than a technical response. A number of institutional arrangements dampen investments in HRH, including a mismatch between officials’ tenure in office and program results, the vertical nature of health programming, the modalities of Overseas Development Assistance (ODA) in health, the structures of the global health community, and the weak capacity in HRH units within Ministries of Health. A major change in policymaking would only occur with a disruption to the political or institutional order.

**Results/conclusions:**

The case study of Ethiopia, who has increased its health workforce dramatically over the last 20 years, disrupted previous institutional arrangements through the power of ideas—HRH as a key intermediate development objective. The framing of HRH created the rationale for the political commitment to HRH investment. Ethiopia demonstrates that political will coupled with strong state capacity and adequate resource mobilization can overcome the institutional hurdles above. Donors will follow the lead of a country with long-term political commitment to HRH, as they did in Ethiopia.

## Introduction

The genesis of the human resources for health (HRH) crisis in African countries is complex and context specific, even if common factors are applicable across the region [1]. Underinvestment in the social sector, which accelerated in the 1980s and 1990s as part of the International Monetary Fund (IMF) and World Bank structural adjustment programs, undermined the health sector [2–6]. The health worker situation in developing countries has deteriorated to crisis levels due to a variety of factors, including political instability and weak health systems, characterized by poor working conditions, while further exacerbated by the migration of health workers to industrialized countries.

The purpose of this paper is to shift the discussion of the solution to the HRH shortage in Africa from the “what” (technical) to the “how” (political). The standard explanation for the health worker shortage is often limited to weak bureaucratic capacity and insufficient resources, which ignores a host of political and economic factors that discourages greater HRH investment. Despite significant attention to the need for more health workers for over a decade, national HRH plans remain under-financed and ultimately not executed and further explanation is needed.

This paper begins with a short description of the extent of the problem. The “Methods” section outlines the parameters of a political economy analysis for HRH which offers a three-level analysis of institutions or structures that shape HRH policy, namely, the political environment, domestic institutions, and international structures. This is followed by a case study of how Ethiopia overcame these barriers to make substantial progress in increasing HRH coverage.

## Background

The lack of health workers in low-income countries has been recognized as a development challenge since the 1970s, beginning with the Alma Ata Declaration or Health for All campaign in 1978 and later nested in broader discussions of structural adjustment in the 1980s and 1990s. HRH rose to the top of the global health agenda through the publication of the Joint Learning Initiative’s report in 2004 and the World Health Report in 2006, which described the extent of the shortage of health workers in a number of countries. Donor agencies responded by targeting increased production of health workers as a key development objective [5, 7–9]. In the re-authorization of the President’s Emergency Plan for AIDS Relief (PEPFAR) in 2008, the legislation set a worldwide target of 140,000 new health workers to be trained by 2013 using PEPFAR funds [8].

In 1978, the World Health Organization (WHO) set a target of one physician per 5000 people in the Alma Ata Declaration/Health for All [10]. In 2006, WHO adopted a threshold of 2.28 skilled health workers per 1000 people; if a country falls below that prescribed threshold, WHO refers to it as an HRH crisis country. There is an empirical basis for this target; it represents the minimum level of coverage needed for 80 % of pregnant women to have adequate ante- and post-natal care and assistance at birth from a skilled health worker, who is defined as a medical officer (physician or doctor), nurse, or midwife (with a 4-year college degree or equivalent) [3].

**Fig. 1:**
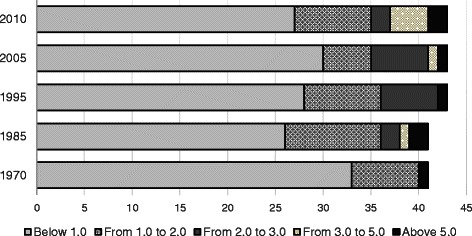
The range of physician density (per 10,000) among African countries, selected years. Eritrea and Namibia were omitted from the 1970 and 1985 results because they were not independent states at this time

According to WHO, there are 57 HRH crisis countries as of 2011 and 37 in Sub-Saharan Africa. The data in Table 1 and Fig. 1 illustrating the number of medical doctors in Africa reveals a legacy of severe shortages. In the face of the persistent lack of health workers in a number of African countries, progress has been slow. In 1970, 33 out of 41 African countries had a physician ratio below one medical doctor per 10,000 population (the physician density), and excluding South Africa (and Namibia, which was part of South Africa until 1993), no African country had a physician density above 2.0 per 10,000 people. By 2010, 27 countries still had a physician density below one per 10,000, and of the 43 countries, only Gabon (2.60), Sudan (2.80), Equatorial Guinea (3.02), Botswana (3.36), Nigeria (3.67), Namibia (4.27), Cape Verde (5.48), and South Africa (7.38) exceeded a physician density of two per 10,000 [11]. Even those countries with seemingly high densities of health workers are beset with HRH mal-distribution between rural and urban areas and between the public and private sectors.

**Table 1:** Ratio of physicians to population (1 MD/10,000), 43 African countries, 1970–2010

Country	1970	1975	1980	1985	1990	1995	2000	2005	2010
Angola	1.16			0.59	0.42	0.77		0.80	1.66
Benin	0.34		0.59	0.73	0.51	0.58		0.40	0.59
Botswana	0.64	1.33	1.23		1.94	2.38	2.88	3.68	3.36
Burkina Faso	0.10		0.18	0.04	0.29	0.34	0.40	0.50	0.47
Burundi	0.17	0.12		0.52	0.58	0.55	0.52	0.28	
Cameroon	0.34	0.72	0.71		0.82	0.74		1.90	0.77
Cape Verde	0.82		1.76	3.29	2.88	1.71		4.90	2.95
Cen. Afr. Rep.	0.23		0.43		0.38	0.35		0.80	0.48
Chad	0.16	0.21		0.26	0.26	0.33	0.25	0.39	
Congo	1.01	1.74	1.19	2.52	2.76	2.51		2.00	0.95
Cote d’Ivoire	0.64			0.71	0.88	0.90		1.20	1.44
Dem. Rep. Congo	0.35	0.36	0.73	0.75	0.66	0.69		1.07	
Equatorial Guinea	0.86				2.81	2.08		3.02	
Eritrea						0.30		0.50	
Ethiopia	0.12	0.12	0.11	0.13	0.31	0.27	0.21	0.26	0.22
Gabon	1.91		4.57	5.18	4.94	2.89		2.92	
Gambia	0.41	0.40		0.81		0.26	0.73	1.09	0.38
Ghana	0.78	0.78		0.67	0.43	0.62	0.76	1.50	0.96
Guinea	0.20		0.22	0.26	1.34	1.30	0.94	1.00	1.00
Guinea-Bissau	0.52		1.41	1.44		1.71	1.66	1.20	0.45
Kenya	1.25	1.27	0.99	1.54	0.45	1.32		1.39	1.81
Lesotho	0.32	0.32		0.71	0.43	0.54		0.49	
Liberia	0.79	0.80	1.06	1.07		0.23		0.30	0.14
Madagascar	0.97	0.96	0.99	1.01	1.20	2.72		2.90	1.61
Malawi	0.13		0.19	0.87	0.22	0.28		0.20	0.19
Mali	0.23	0.23	0.39	0.39	0.51	0.43	0.44	0.80	0.83
Mauritania	0.56	0.55		1.13	0.63	1.38		1.10	1.30
Mozambique	0.53		0.25	0.21	0.12		0.24	0.29	0.26
Namibia					2.31	2.63	2.95	3.00	3.74
Niger	0.17	0.16		0.25	0.18	0.23	0.18	0.20	0.19
Nigeria	0.50	0.49	1.13	1.92	1.92	1.92	2.69	2.80	3.95
Rwanda	0.17	0.17	0.32	0.27	0.40		0.56	0.24	0.56
Senegal	0.63	0.61	0.79	0.77	0.55	0.75	0.55	0.60	0.59
Sierra Leone	0.56	0.54	0.58	0.75		0.73		0.30	0.22
Somalia	0.37	0.38	0.46	0.71		0.41		0.35	0.35
South Africa		5.39	5.51	6.13	6.24	5.93	7.70	7.70	7.58
Sudan	0.68	0.68	1.10	0.96		0.97	1.58	2.20	2.80
Swaziland	1.24			0.53	1.08	1.51	1.76	1.60	1.70
Tanzania	0.44	0.44		0.35	0.40	0.41	0.08	0.08	0.08
Togo	0.35	0.35	0.55	1.01	0.88	0.76	0.40	0.40	0.53
Uganda	1.08	1.08	0.47	0.44	0.45	0.40	0.47	1.17	1.17
Zambia	0.73	0.74	0.76	1.41	0.92	0.69		0.88	0.66
Zimbabwe	1.62	1.58	1.61	1.62	1.35	1.39	0.57	1.60	0.62

Source: World Development Indicators [11]

Notwithstanding their severe shortages, most HRH crisis countries have made little progress in increasing the production of new health workers. Many have implemented some form of task-shifting—a policy that expands the scope of work of lower skilled health workers to cover activities that higher skilled health workers would usually perform [12]. An example of this is nurse-initiated management of anti-retroviral therapy (NIMART), which began in the Southern African region, necessitated by the shortage of physicians [13]. Many HRH crisis countries have implemented new recruitment and retention schemes, with varying success [14, 15]. Task-shifting and retention programs have extensive literatures, and they are outside the scope of this paper. The production of more health workers is necessary, and the failure of many African countries to do so will be the focus of the political economy analysis below.

## Methods

The rationale for a political economy analysis of HRH stems from a recognition that the solution to the shortage of health workers across Africa involves more than a technical response. The problem consists in how policy is made, how leaders are accountable, how WHO and foreign donors encourage (or distort) health policy, and how development objectives are prioritized in these countries. Fundamentally, this is a political process, which presents constraints and opportunities for potentially effective technical solutions to be adopted and implemented. In this article, a series of assumptions characterize the policy process broadly [16–18]:

- Ambiguity—there are multiple ways to frame any policy problem.
- Competition—few problems receive substantial attention and move to the top of the agenda.
- Bounded rationality—politicians and bureaucrats must make decisions based on limited or manipulated information.
- Interests—all politicians and bureaucrats have personal and professional ones.
- Incentives—institutions, or the rules of the game, and resources create incentives for all politicians and bureaucrats, which influences and ultimately shapes their behavior.
- Disruption—only upheaval in the political or institutional order leads to a change in the policy change, which might be an election, a new technology, or a powerful idea.

A political economy analysis was adapted to explain the lack of progress in the HRH space, namely, the inadequate production of health workers. This approach applied economic thinking to politics framed by the assumptions above [16, 19]. This institutional political economy approach has become a common tool for understanding the political constraints in development and policymaking generally [18, 19]. The political economy analysis in this paper is applied to a literature review and later on Ethiopia as a case study.

This article adopts a broader institutional political economy approach to analyze the HRH crisis in Africa by examining multiple levels—the political environment, domestic institutions, and international structures. This paper applies the key tenets of a political economy analysis to the lack of progress in increasing health worker density with HRH crisis countries in Africa. The political environment refers to the competition to frame the development agenda, the influence of civil society, and the interests of stakeholders. Domestic institutions refer to the capacity of the public sector and the incentives driving policymaker actions. International structures include the role of donors, global campaigns, and WHO guidelines and instruments, including the WHO Global Code of Practice on International Recruitment of Health Personnel. The analysis of these three levels reveals opportunities to encourage more action within the HRH space and specifically the increased production of health workers.

## Results and discussion

### Political environment

#### Multiple stakeholders in the HRH sector

In most African countries, at least a dozen stakeholders occupy the HRH policy space: Ministries of Education, Health, Finance, and Public Service; training institutions (public and private); professional boards and associations; WHO; and international donors [20]. With all those actors, it is difficult to assign blame (or credit) to an individual for the failure (or success) to meet adequate staffing levels. The multiple stakeholders in the HRH space effectively insulate politicians from blame and prevent them from taking credit.

If a politician or bureaucrat sought to expand HRH production, agreement among all parties would be needed. A proposal for greater HRH investment would produce immediate rivalry or conflict between or among ministries. Not only would a consensus need to be reached for an increased production of health worker, the public service ministry would also have to accede to the budget allocation for additional posts, and the finance ministry would have to guarantee adequate funding for such expansion in numbers. The number of players with a veto in the HRH space transforms HRH into a collective action problem.

#### Limited resources

Decisions are often resource-driven, and politicians in developing countries face a myriad of social demands, from agriculture and food security to transportation, infrastructure, education, and health. Despite high disease burdens, only a handful of African nations (Rwanda, Botswana, Burkina Faso, Zambia, Mali, and Niger) have met the Abuja Declaration (2001) that set a target of 15 % of government revenue to be directed to health [21]. In the contentious world of agenda setting, health ministers and their teams seem to make unconvincing arguments to their finance peers or their bosses (the national executive), or they fail to frame health as value for money.

Policymakers often view expenditure on health as consumption of resources, rather than as an investment, and this is evident in national strategies. Politicians also need to deal with their national disease burden, with health budgets often targeting vertical programs: HIV, TB, malaria, or maternal health (e.g., a campaign to end obstetric fistulas). The prioritization of vertical programs over HRH production and, more broadly, health system strengthening plague Ministries of Health at all levels of development [22] with only a small fraction of the US$ 6 trillion spent annually on health around the world going to pre-service education [23, 24].

#### Weak civil society pressure

Citizens usually do not place blame on politicians for a clinic that is not properly staffed because they are not necessarily aware of what a fully staffed clinic would look like. Even if they did, the link between the under-staffed clinic and the corresponding decisions by politicians is not immediately clear [25], and there is little confidence in many African countries that structural problems will be addressed [26]. Information on national health worker density is often unavailable, and when it is, it is often outdated. The lack of a clear causal arrow between health worker density and health outcomes and the paucity of information makes it difficult for citizens to mobilize around HRH issues.

While the role of civil society engagement in policymaking and implementation has been highlighted through the development process for the WHO Code of Practice, this level of engagement has been missing after adoption of the Code and has been partly held responsible for the poor uptake, implementation, and impact of the Code [27]. Despite the best efforts of NGOs like the Frontline Health Workers Coalition, the absence of advocacy from civil society, both domestically and internationally, encourages the malaise towards health workforce issues.

### Domestic institutions

#### Mismatch between electoral cycles/tenure in office and programmatic results

Politicians and bureaucrats need to claim credit for some achievement or success to further their careers, and they face substantial professional disincentives for HRH investment. Longer-term investments are not attractive to many politicians or ministers, especially if they continue after the end of their mandate [28]. It is nearly impossible for a politician or minister to claim credit for a new school or hospital if it was completed after their tenure. The time needed to compose a plan for health worker production might take up to 2 years, and production of one highly skilled health worker might take 4 to 6 years. Governments seeking to increase production will not see the evidence of their strategies for at least 6 to 10 years. Tenure of most ministers is less than 5 years, which entices them to adopt policies and strategies which deliver more immediate results [29].

#### Weak capacity in the bureaucracy (specifically the HRH unit)

One of the greatest impediments to the production of more health workers is the lack of bureaucratic capacity within national Ministries of Health. The HRH units are hampered by the lack of a clear mandate, poor technical skills, and weak coordinating powers to utilize expertise from within the ministry or broader government where they exist. Of the 57 HRH crisis countries in Africa, 31 of them had HRH strategic plans by 2009. International consultants wrote most of these plans, and the subsequent dependence on international consultants means that the plan is not country-owned and little skill transfer occurs. After the consultants leave, the staff in the HRH units neither have the acumen to implement the plans nor the skills to monitor the activities of the plan [30–32].

Part of country ownership is that policy entrepreneurship is required to champion a policy change as intensive as the implementation of an HRH plan, without which, the domestic policy entrepreneurs or champions do not exist. The lack of policy champions or entrepreneurs for HRH has been one of the reasons that the commitment of resources required for a major scale-up in production has not occurred. John Kingdon describes a policy entrepreneur who “lie(s) in wait in and around government with their solutions at hand, waiting for problems to float by to which they can attach their solutions, waiting for a development in the political stream they can use to their advantage” [17]. Within many Ministries of Health across Africa, few bureaucrats have the technical acumen to be effective policy champions. Those that do are often constrained by office politics [29, 32]. The status of the position of the HRH director widely varies in ministries, and the role is not usually considered a prestigious or high-profile post. The position of a director or office chief is tied to a Principal Secretary (PS) or Director-General (DG), who is usually the second in line under the Minister, and does not want to be viewed as disruptive or antagonistic [30].

### International structures

#### The role of Overseas Development Assistance (ODA)

ODA might impede domestic efforts to increase HRH production. Many donors provide assistance on an annual basis, which renders long-term planning impossible. Aid agencies drive Ministries of Health towards treatment of specific diseases (e.g., HIV and TB) in part because they are anxious to take credit for their assistance in saving lives to placate various stakeholders (from parliaments or legislatures) who determine their annual budgets [7, 22, 24]. Other health issues take precedent due to their urgency and relative ease to solve.

#### The Millennium Development Goals (MDGs)/Sustainable Development Goals (SDGs)

While the MDGs did pressure developing countries and the donor community to mobilize towards achieving important targets in global health, the MDGs and the SDGs that have followed suffer from an emphasis on final targets without an adequate focus on intervening or secondary targets that influence primary health outcomes. Traditionally, maternal mortality has been considered a robust proxy for the strength of a health system, but HRH issues like the production of more health workers were largely absent from the discussion of MDG 5 [33, 34]. The post-2015 global development agenda might be another missed opportunity as emerging issues, such as climate change, sanitation, water, and gender-based violence, crowd out the HRH issue.

#### The vertical structures within global health

When donors want to address an issue in global health, they often decide that it will be through an alliance independent of the WHO, e.g., HIV/AIDS (UNAIDS was established as a separate entity), TB (STOP TB), and the war against immunizable diseases (Global Alliance for Vaccines and Immunization (GAVI)). In HRH, this took the form of the Global Health Workforce Alliance (GHWA). Notwithstanding the successes of these parallel structures, the pattern is clear: once WHO identifies a priority area, there is a tendency to establish a separate structure to work on the problem. Much of the justification speaks to the rigidity of the WHO system, which in most cases means that donors want to retain absolute control of their resources through these structures [35–38].

GHWA did provide resources to countries on a wide range of technical aspects of HRH and provided a forum that mobilized some civil society groups and some ODA for HRH. From 2009 to 2014, PEPFAR spent US$ 2.5 billion on HRH and established a target of 140,000 new health workers to be trained by 2013 [8]. Despite these successes, GHWA did not produce an adequate level of resource mobilization from domestic and international sources to address the shortage of health workers in the 37 African crisis countries. GHWA also might have had the unintended consequence of isolating HRH and limiting the potential linkages to other health issues like maternal and child mortality.

#### The WHO Global Code of Practice on International Recruitment of Health Personnel

The Code was adopted after a protracted period of negotiation in May 2010. The guiding principles of the WHO Code include ethical international recruitment of health workers, a focus on the health system in the context of HRH sustainability, fair treatment of migrant health workers, support to developing countries and especially HRH crisis countries, data gathering on HRH migration, and information exchange [39, 40]. With the Code came expectations that countries would make adequate investments in their HRH production and retention and would strengthen HRH governance structures. The Code also called for bilateral and multi-lateral arrangements that recognize the contexts of different countries, e.g., bilateral agreements for one country to recruit health workers in another country.

Brain drain largely overshadowed the broader discussion at the World Health Assembly, which the Code addressed through three principles: (1) the right of a health worker to migrate is a human right; (2) countries are free to accept migrant health labor, but they should not recruit actively for it; and (3) labor-receiving countries have no responsibility to compensate labor-sending countries for their loss of labor [39]. The final point continues to be particularly contentious among HRH crisis countries. The Code was viewed not as a path to deal with the HRH shortage systematically but rather as an unfair compromise in which the negotiation was a zero-sum game that labor-receiving countries won [41, 42]. As a result, the Code was never the comprehensive framework or the disruptive force for HRH mobilization that it was intended to be.

Within development circles, the issue of relevance became the most severe criticism of a political economy approach. In 2011, a World Bank report summarized: “While political economy studies appear to be useful in providing insight into the country context and facilitate a better and quicker understanding of the realities on the ground, translating political economy analysis into action remains a challenge” [19]. If a change is to occur in the policy agenda, it usually happens due to a disruption in the political or institutional arrangements. This tumult in the status quo might take the form of a regime change, an election result, an emerging idea, or a new technology [17, 18]. This paper offers Ethiopia as an example of how HRH crisis countries might create the disruption needed to overcome the political and structural hurdles for greater health worker coverage.

### The Ethiopian experience

Upon the release of the 2006 WHO Report, Ethiopia had among the lowest health worker densities in the world. Ethiopia has made substantial progress in improving health worker coverage, even if it failed to increase the production of physicians as shown in Table 1. Ethiopia increased its health worker density (including all cadres) per 1000 population from 0.43 in 2003 to 0.86 in 2008, and Ethiopia expanded its enrollment of medical doctor trainees from below 300 in 2005 to 3100 in 2012 [43]. The Ethiopian example shows that opportunities do exist in overcoming these political economy barriers.

#### Political will

In the mid-1990s, Ethiopia committed to a long-term (20 plus years) strategy to address health service provision. At a very low level of health worker density, Ethiopia recognized that progress in HRH would require decades to improve service coverage. In the face of a crowded development agenda, HRH needed to be linked with other development objectives to be prioritized. The Health Sector Development Program (HSDP) is a multiple-phase plan to improve health service coverage and provision in which HRH planning was explicitly part of all four of its iterations [43, 44].

Ethiopia’s HRH plan within HSDP phase III from 2005 to 2009 was designed to address coverage issues first through the deployment of health extension workers (community health workers) and only later to increase the stock of more skilled health cadres. Ethiopia also introduced a new cadre of health workers, the health officer, to provide basic health services and to manage the expansion of its public health service. In 2009, the Ministry revealed its HRH Strategy 2020, a plan that addressed higher skilled health cadres, in particular medical doctors, whose stock was planned to rise from less than 5000 in 2009 to 50,000 trained by 2020. Ethiopia is on track to meet this target, which is to be reflected in its number of physicians in 2015 [43].

As a stepping stone to better service provision, the health workforce was prioritized in achieving better health outcomes. This framing of HRH formed the basis of the political will to sustain the program over many years. The power of the idea that HRH was a crucial element in improved health services disrupted the previous order by changing the priorities within the development agenda. The national long-term commitment to HRH resulted in access to resources with domestic stakeholders forced to work within the directive of the HSDP.

#### State capacity

One of the major obstacles for HRH crisis countries to raise their health worker density is the weak capacity of the government to plan and coordinate effectively. During the process of expanding the health workforce, Ethiopia implemented Business Process Reengineering (BPR) at all its federal and regional governing departments and units. BPR was a program to introduce results-based management to the public sector. Despite mixed results, BPR strengthened capacity in the government overall. The Ministry of Health demonstrated its increased capacity in the quality of its strategic planning in health and HRH, its donor coordination, and its management of large sums of ODA.

As HRH was emerging as a global issue in the first decade of the 2000s, nearly every HRH crisis country in Africa wrote a strategic HRH plan to address its own health worker shortage. Many national HRH plans suffered from similar problems: over-ambitious targets, disruptions (or even abandonment) due to leadership changes, little political support, and weak resource mobilization. Ethiopia differed in that it wrote its first HRH plan in 1995, a decade before the publication of World Health Report 2006. The plan included a thorough costing of all activities; it was monitored rigorously and evaluated independently; and stakeholders, external and internal, considered the plans to be country-owned. The successive HRH plans became an advocacy tool domestically and globally, particularly for donors [45–48].

Ethiopia has had authoritarian governance for the last two decades, and there might be a temptation to argue that authoritarianism might be good for development and HRH specifically.

Without the pressure from civil society or an electorate in a democratic society, as the argument follows, bureaucrats are free to act and implement effective development policies that might be unpopular in the short-term. Another interpretation of this argument is skeptical that democratic rule can promote good governance in new regimes in Africa [49]. This argument might seem compelling, but there are reasons to discount it. Many HRH crisis countries have authoritarian regimes, and they have failed to achieve any improvement in health worker density. Democratic regimes are likely to have better development outcomes, because mechanisms of accountability encourage politicians and bureaucrats to act on behalf of the common good [50]. In the case of Ethiopia, there is no way to know if the policy trajectory towards better health outcomes and increased HRH coverage would have been different or not under democratic rule.

#### Resource mobilization

Ethiopia has received a large amount of ODA in health. In current, purchasing power parity (PPP) terms, Ethiopia’s total per capita health spending nearly tripled from US$ 28 in 2005 to US$ 69 in 2013, largely due to ODA. From 2003 to 2009, The Global Fund to Fight AIDS, Tuberculosis and Malaria (GFATM) gave Ethiopia over US$ 3 billion, and from 2007 to 2014, PEPFAR provided US$ 2 billion [45] with US$ 165 million specifically earmarked for HRH production between 2009 and 2014 [46]. While other countries, especially those with generalized HIV epidemics, experienced similar growth in health spending, a significant proportion of the ODA received by Ethiopia was allocated to support HRH production, which was not the case in other HRH crisis countries [24, 47, 48].

This was a direct result of the strategic health plans adopted and implemented by the Ethiopian government which donors could support and convince their own stakeholders of the potential impact. Even though donors have their own interests and face domestic pressures regarding ODA, they are also open to recipient country initiatives. Despite the inconsistency of annual ODA flows and large off-budget funding in vertical programs, Ethiopia channeled their assistance into increasing health worker density through thoughtful planning and coordination with donors. Its Ministry of Finance and Economic Development designed the Aid Management Platform to harmonize the various streams of ODA, which has become a best practice for donor coordination [44, 47]. A government official from Ethiopia commented, “What development partners need is openness, accountability and results. This is why support from development partners, whether bilateral or multi-lateral, is increasing. They are all working with the policy and program frameworks prepared by the government through broad consultations with stakeholders” [44].

## Conclusions

Despite a global recognition of the gravity and urgency of health worker shortage in Africa, little progress has been achieved to improve health worker coverage in many of the African HRH crisis countries generally. Powerful political and institutional incentives push stakeholders at the domestic and international levels not to invest in HRH. The status quo of institutional arrangements needs to be changed for new policy choices to reach the top of the agenda, and ideas have the power to be the earthquake to disrupt the previous rules of the game. Good governance and some degree of bureaucratic capacity alone do not ensure a successful HRH plan. We argue that political will, in the form of a long-term commitment to HRH, is essential to mobilize internal and external resources. We also contend that this political commitment to HRH was the product of framing—policy entrepreneurs successfully tied more health workers to better health services for more Ethiopians.

Donors play a vital role in the HRH space, but they should not be leading the charge. The case of Ethiopia shows that if recipient countries present a reasonable national HRH plan to donors, they are willing to support it through ODA, as long as the donors receive attribution for results that they can present to their legislatures or parliaments. The WHO Global Code of Practice on International Recruitment of Health Personnel was a missed opportunity to create a regime of monitoring of health worker density, similar to the Millennium Development Goals. The monitoring of HRH progress remains a significant challenge for many HRH crisis countries.

The WHO target of 2.28 skilled health workers per 1000 people might require 20 years or more for some African countries to attain, and the project would need to outlive individual directors of HRH units, Ministries of Health, and heads of state. Consensus politics is difficult to practice and maintain in any country, and it has been elusive in most of the HRH crisis countries in Africa. Even though Ethiopia has enjoyed initial success, it requires at least another decade to reach the WHO health worker target. While Ethiopia provides an example of a country that is successfully navigating its way past the very same political economy challenges facing other African HRH crisis countries, it is abundantly clear that there remains no quick fix for the HRH shortage.
